# Supplementary material for: Identifying daily-living features related to loneliness: A causal machine learning approach
Source: PLoS One. 2025 Dec 17;20(12):e0336287. doi: 10.1371/journal.pone.0336287 (PMC12711064; doi:10.1371/journal.pone.0336287)
Supplement: S1 File. — Full List of Features. Table A. List of features from Samsung watch; Table B. List of features from OURA ring; Table C. List of features extracted from smartphone. S1 Fig. Sensitivity Analysis. Fig A. ATE Estimates and Confidence Intervals by Feature Using KNN and MI Imputation; Fig B. Absolute ATE Difference Between KNN and Multiple Imputation. S1 Text. Inclusivity in Global Research Questionnaire. (ZIP) [file pone.0336287.s001.zip › S1_Fig.docx]

**S1 Fig. Sensitivity Analysis**


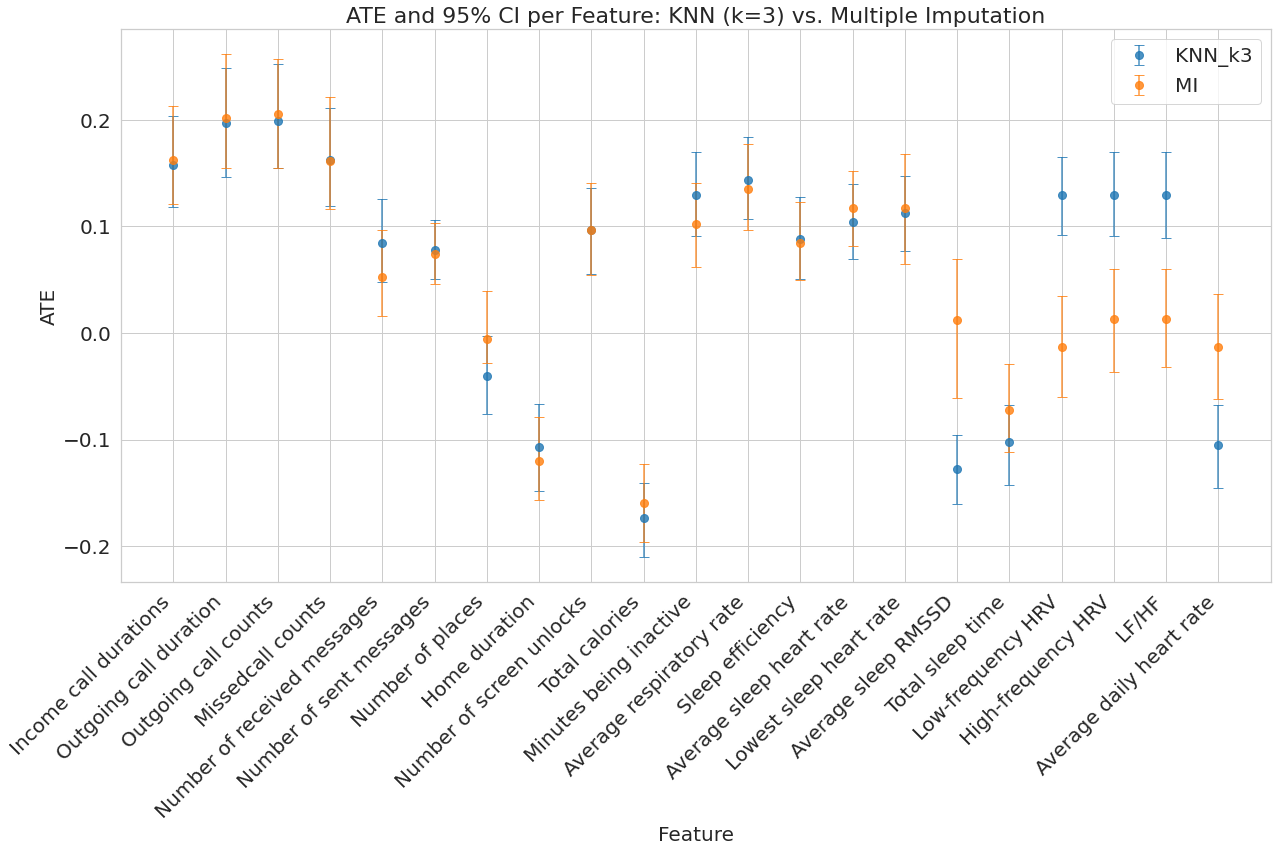


Fig A. ATE Estimates and Confidence Intervals by Feature Using KNN and MI Imputation


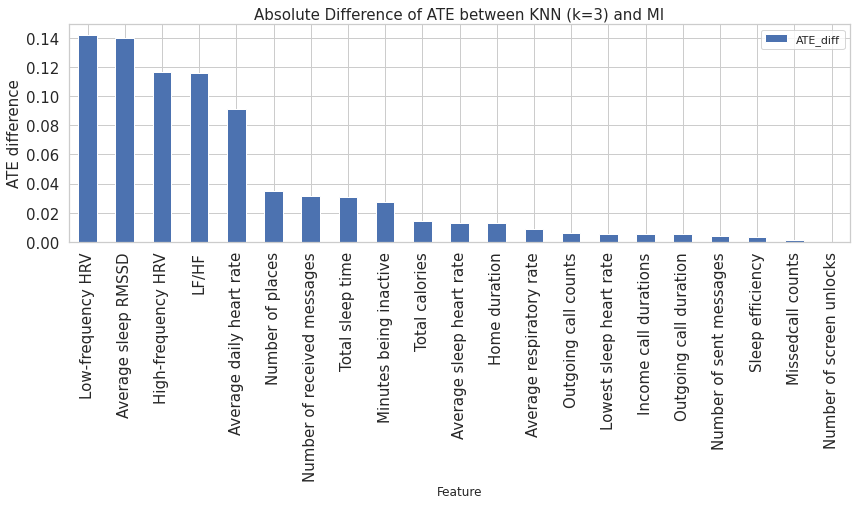


Fig B. Absolute ATE Difference Between KNN and Multiple Imputation
