# Supplementary material for: Identifying daily-living features related to loneliness: A causal machine learning approach
Source: PLoS One. 2025 Dec 17;20(12):e0336287. doi: 10.1371/journal.pone.0336287 (PMC12711064; doi:10.1371/journal.pone.0336287)
Supplement: S1 File. — Full List of Features. Table A. List of features from Samsung watch; Table B. List of features from OURA ring; Table C. List of features extracted from smartphone. S1 Fig. Sensitivity Analysis. Fig A. ATE Estimates and Confidence Intervals by Feature Using KNN and MI Imputation; Fig B. Absolute ATE Difference Between KNN and Multiple Imputation. S1 Text. Inclusivity in Global Research Questionnaire. (ZIP) [file pone.0336287.s001.zip › S1_Table.docx]

**S1 Table. Full List of Features**

Table A. List of features from Samsung watch [1,2]

| **Parameter** | **Description** |
| --- | --- |
| HRV_MeanNN | The mean of the RR intervals. |
| HRV_SDNN | The standard deviation of the RR intervals. |
| HRV_SDANN1 | The standard deviation of average RR intervals extracted from 1-minute segments of time series data |
| HRV_SDNNI1 | The mean of the standard deviations of RR intervals extracted from 1-minute segments of time series data |
| HRV_RMSSD | The square root of the mean of the squared successive differences between adjacent RR intervals. |
| HRV_SDSD | The standard deviation of the successive differences between RR intervals. |
| HRV_CVNN | The standard deviation of the RR intervals (SDNN) divided by the mean of the RR intervals (MeanNN). |
| HRV_CVSD | The root mean square of successive differences (RMSSD) divided by the mean of the RR intervals (MeanNN). |
| HRV_MedianNN | The median of the RR intervals. |
| HRV_MadNN | The median absolute deviation of the RR intervals. |
| HRV_MCVNN | The median absolute deviation of the RR intervals (MadNN) divided by the median of the RR intervals (MedianNN). |
| HRV_IQRNN | The interquartile range (IQR) of the RR intervals. |
| HRV_pNN50 | The proportion of RR intervals greater than 50ms, out of the total number of RR intervals. |
| HRV_pNN20 | The proportion of RR intervals greater than 20ms, out of the total number of RR iintervals. |
| HRV_HTI | The HRV triangular index, measuring the total number of RR intervals divided by the height of the RR intervals histogram. |
| HRV_TINN | The baseline width of the RR intervals distribution obtained by triangular interpolation, where the error of least squares determines the triangle. It approximates the RR interval distribution. |
| HRV_LF | The spectral power of low frequencies (0.04 to 0.15 Hz). |
| HRV_HF | The spectral power of high frequencies (0.15 to 0.4 Hz). |
| HRV_VHF | The spectral power of very high frequencies (0.4 to .5 Hz). |
| HRV_LFHF | The ratio obtained by dividing the low frequency power by the high frequency power. |
| HRV_LFn | The normalized low frequency, obtained by dividing the low frequency power by the total power. |
| HRV_HFn | The normalized high frequency, obtained by dividing the low frequency power by the total power. |
| HRV_LnHF | The log transformed HF. |
| HRV_SD1 | Standard deviation perpendicular to the line of identity. It is an index of short-term RR interval fluctuation |
| HRV_SD2 | Standard deviation along the identity line. Index of long-term HRV changes. |
| HRV_SD1SD2 | Ratio of SD1 to SD2. Describes the ratio of short term to long term variations in HRV. |
| HRV_S | Area of ellipse described by SD1 and SD2 (pi * SD1 * SD2) |
| HR | Heart rate |

Table B. List of features from OURA ring [3]

| **Category** | **Feature** |
| --- | --- |
| Physical Activity | Average MET level during the whole day |
|  | Energy consumption caused by the physical activity of the day in kilocalories |
|  | Total energy consumption during the day including Basal Metabolic Rate in kilocalories |
|  | The activity intensity level |
|  | Number of minutes during the day with high intensity activity (e.g. running) |
|  | Number of inactive minutes (sitting or standing still, average MET level of the minute between 1.05 and 2) during the day |
|  | Number of minutes during the day with low intensity activity (e.g. household work, average MET level of the minute between 2 and age dependent limit) |
|  | Number of minutes during the day with medium intensity activity (e.g. walking). The upper and lower MET level limits for medium intensity activity depend on user's age and gender |
|  | Average MET level for each minute of the activity period, starting from 4 AM local time |
|  | Total MET minutes accumulated during high intensity activity minutes of the day |
|  | Total MET minutes accumulated during inactive minutes of the day |
|  | Total MET minutes accumulated during low intensity activity minutes of the day |
|  | Total MET minutes accumulated during medium intensity activity minutes of the day |
|  | Number of minutes during the day when the user was not wearing the ring |
|  | Number of minutes during the day spent resting i.e. sleeping or lying down (average MET level of the minute is below 1.05) |
|  | Total number of steps registered during the day |
| Sleep | Total amount of awake time registered during the sleep period |
|  | Average respiratory rate |
|  | Local time when the sleep period ended |
|  | Sleep period end time difference from long-term sleep end time average |
|  | Local time when the sleep period started |
|  | Sleep period start time difference from long-term sleep start time average |
|  | Total amount of deep (N3) sleep registered during the sleep period |
|  | Sleep efficiency (i.e., the percentage of the sleep period spent asleep) |
|  | Average heart rate during sleep |
|  | The lowest heart rate during sleep |
|  | Total amount of light (N1 or N2) sleep registered during the sleep period |
|  | Detected latency from bedtime_start to the beginning of the first five minutes of persistent sleep |
|  | Total amount of REM sleep registered during the sleep period |
|  | Number of restless periods during sleep |
|  | The average HRV calculated with rMSSD method |
|  | Skin temperature deviation from the long-term temperature average |
|  | Skin temperature deviation from environment |
|  | Total amount of sleep registered during the sleep period |

Table C. List of features extracted from smartphone [4]

| **Category** | **Feature** |
| --- | --- |
| Call | Income call durations |
|  | Outgoing call duration |
|  | Income call counts |
|  | Outgoing call counts |
|  | Missed call counts |
|  | Voicemail counts |
| Notification | Number of notifications from applications type: Productivity |
|  | Number of notifications from applications type: Photography |
|  | Number of notifications from applications type: Communication |
|  | Number of notifications from applications type: Lifestyle |
|  | Number of notifications from applications type: Social |
|  | Number of notifications from applications type: Shopping |
|  | Number of notifications from applications type: Health & Fitness |
|  | Number of notifications from applications type: Entertainment |
|  | Number of notifications from applications type: Music & Audio |
| Messages | Number of received messages |
|  | Number of sent messages |
| Battery | Number of battery charger plugins |
| Screen activity | Number of screen off |
|  | Number of screen on |
|  | Number of screen locks |
|  | Number of screen unlocks |
| Location | Variance of latitude |
|  | Variance of speed |
|  | Mean of speed |
|  | Number of places |
|  | Home duration |
|  | Outdoor duration |
|  | Mean of outdoor duration |
|  | Standard deviation of outdoor duration |
|  | Type of the place with longest duration other than home |
|  | Total travel distance |

**Reference:**

[1]. Feli M, Kazemi K, Azimi I, Wang Y, Rahmani AM, Liljeberg P. End-to-End PPG Processing Pipeline for Wearables: From Quality Assessment and Motion Artifacts Removal to HR/HRV Feature Extraction. 2023 IEEE International Conference on Bioinformatics and Biomedicine (BIBM). 2023;1895–1900. doi:10.1109/BIBM58861.2023.10385998

[2]. Samsung. Galaxy Watch Active2 | Samsung Suomi. Samsung Finland. Available from: https://www.samsung.com/fi/watches/galaxy-watch-active2/

[Accessed 14 Jan 2025].

[3]. Oura. Oura Ring. Smart Ring for Fitness, Stress, Sleep & Health. Oura Ring. Available from: https://ouraring.com

[Accessed 14 Jan 2025].

[4]. van Berkel N, D’Alfonso S, Kurnia Susanto R, Ferreira D, Kostakos V. AWARE-Light: A smartphone tool for experience sampling and digital phenotyping. Pers Ubiquitous Comput. 2023;27(2):435–445. doi:10.1007/s00779-022-01697-7
