# Supplementary material for: Identifying daily-living features related to loneliness: A causal machine learning approach
Source: PLoS One. 2025 Dec 17;20(12):e0336287. doi: 10.1371/journal.pone.0336287 (PMC12711064; doi:10.1371/journal.pone.0336287)
Supplement: Attachment — Submitted filename: Response to Reviewers_0708.docx [file pone.0336287.s004.docx]

Response to Reviewers – Manuscript ID: PONE-D-25-06716

Title: Identifying Daily-Living Features Related to Loneliness: a Causal Machine Learning Approach

Dear Editor and Reviewers,

We thank you for the opportunity to revise our manuscript “*Identifying Daily-Living Features Related to Loneliness: a Causal Machine Learning Approach*” (PONE-D-25-06716), for consideration for publication in *PLOS One.* We are delighted to receive feedback from the Reviewer, as well as to address their remaining comments. In this letter we provide a point-by-point response to their comments.

**Reviewer #1: Dear authors,**

I would like to thank you for revising your manuscript in such a thoughtful manner. I particularly appreciate your clear explanations on PPG signals and the overall improvements in the writing flow! Also, great news on the data availability – thank you and well done.

Two issues remain open for me.

1. The issue of the nursing expert / health science researchers remains a little unresolved to me. The term "nursing experts" has been changed to "health science researchers" (except in section "Derived Physiological and Behavioural Features").

But just changing the label for the experts does not fully resolve the issue: First of all, health science researchers is just as vague as nursing experts. I think the more important detail to disclose is that the two experts are not independent experts, but members of your team. This should be addressed in the paper for transparency.

1.1 Are they also co-authors of this paper? If so, that should be disclosed in the paper as well.

1.2 In the response to the review you also disclosed the number of experts (n = 2), but in the paper state "a team of" [...] experts. It seems a bit misleading to refer to two people as a team, as it makes it sound as if you had an entire team of independent experts evaluate your work.

Please note that I do not wish to torture you with these seemingly minor concerns. As researchers, we are often constrained by limited resources and have to rely on the help and support of close colleagues and team members. This is not an issue in itself, but should be disclosed honestly and transparently.

- Thank you for these comments and your kind clarification! We fully agree and appreciate your suggestion regarding the transparency. In the revised manuscript, we have addressed the concerns as follows:

- We now clearly state that the two individuals who reviewed the causal graphs and contributed domain-specific insight are both co-authors of the paper and members of the research team (a Professor and an Assistant Professor in Nursing Science).
- We have removed vague terms such as “nursing experts” and “a team of experts” throughout the manuscript and replaced them with a clear description of their roles. Specifically:
  - In the *Materials and Methods/Measures*/*Derived Physiological and Behavioral Features/paragraph 2* and *Data Analytic Plan/Causal machine learning/Causal discovery/paragraph 2*, we revised the text to state: “Two members of the research team (a Professor and an Assistant Professor in Nursing Science, both co-authors of this paper) reviewed the…”
- In the *Discussion/Implications for future research*, we added the following statement: “Furthermore, the feature selection in this study was conducted by two co-authors from the research team, rather than independent experts. This may introduce bias and limit the objectivity of the results, reducing their generalizability without external validation.”

2. One methodological note: The interpretation of the ATEs in Table 2 appears to rely heavily on statistical significance (p-values), even in cases where the effect size is close to zero. For example, the ATE for Number of Places is -0.041. While statistically significant, it can be questioned whether the effect is practically relevant at all.

Yet, the authors put effort into interpreting the contradiction of this significant ATE with the somewhat contradictory significant negative effect of Home Duration.

In general, the sizes of the effects were not addressed anywhere in the paper, if I recall correctly.

- Thank you for your observation. We agree that statistical significance alone does not guarantee practical or clinical relevance, and that small effect sizes (e.g., the ATE for Number of Places (-0.041)) should be interpreted with caution. We addressed the concerns in the revised manuscript. Specifically:

- We have added 95% confidence intervals for all ATE estimates in Table 2, and included error bars in Figure 1. These additions provide readers with clearer insight into the precision and variability of our estimates. The confidence intervals can help the readers judge the robustness and potential practical relevance of each finding beyond only p-values.
- In the *Discussion/paragraph 2*, where we discussed the Home Duration and Number of Places, we added the following statement: “However, it is worth noting that the estimated average treatment effect (ATE) for some features—such as Number of Places—was relatively small (i.e., -0.041), despite reaching statistical significance. This suggests that while the association may be consistent, its practical impact on loneliness is limited. Therefore, interpretations should be made cautiously, considering both the effect size and the broader behavioral context.”
- In the *Discussion/Implications for future research/paragraph 2*, we emphasized: “Some small but statistically significant ATEs may reflect consistent but subtle behavioral signals, rather than large-scale intervention targets.”
